# Supplementary material for: Scanning Ion-Conductance Microscopy for Studying Mechanical Properties of Neuronal Cells during Local Delivery of Glutamate
Source: Cells. 2023 Oct 11;12(20):2428. doi: 10.3390/cells12202428 (PMC10604991; doi:10.3390/cells12202428)
Supplement: Supplementary file 1 [file cells-12-02428-s001.zip › cells-2580898-supplementary.pdf]

Supporting Information for

Scanning Ion-Conductance Microscopy for Studying Mechanical Properties of Neuronal Cells during Local Delivery of Glutamate

Vasilii Kolmogorov [1,2], Alexander Erofeev [1], Alexander Vaneev [1,2], Lyubov Gorbacheva [3,4], Dmitry Kolesov [5],
Natalia Klyachko [2], Yuri Korchev [6,7] and Petr Gorelkin [1,*]

1 Research Laboratory of Biophysics, National University of Science and Technology "MISIS", Moscow 119049, Russia; vskolmogorov@gmail.com (V.K.); erofeev.as@misis.ru (A.E.); vaneev.aleksandr@gmail.com (A.V.)
2 Faculty of Chemistry, Lomonosov Moscow State University, Moscow 119991, Russia; nlklyachko@gmail.com
3 Faculty of Biology, Lomonosov Moscow State University, Moscow 119991, Russia; gorbi67@mail.ru
4 Faculty of Biomedicine, Pirogov Russian National Research Medical University, Moscow 117997, Russia
5 Research Laboratory of SPM, Moscow Polytechnic University, Moscow 107023, Russia; dmitry.v.kolesov@gmail.com
6 Department of Medicine, Imperial College London, London SW7 2BX, UK; y.korchev@imperial.ac.uk
7 Nano Life Science Institute (WPI-NanoLSI), Kanazawa University, Kanazawa 920-1192, Japan
* Correspondence: peter.gorelkin@gmail.com

To estimate the electroosmotic flow rate from the nanopipette, we used the formula(Babakinejad et al. Anal. Chem., 2013) to calculate the number of moles of sodium glutamate flowing out of the nanopipette (S1):

$$Q_{\Delta\Psi} = \mu_{eo}\pi R_0 \tan(\theta)\Delta\Psi \quad \text{(S1)}$$

there μ_{eo} – electroosmotic mobility, R_0 – radius of nanopipette (R_0 = 50 nm), θ — half-cone angle of the inner wall, ΔΨ- potential difference. The electroosmotic mobility could be determined by (S2)

$$\mu = -\frac{\varepsilon_r \varepsilon_0 \zeta}{\eta} \quad \text{(S2)}$$

there ζ – dzeta potential (ζ = −20 mV for SiO_2 surface), η - the viscosity of the solution inside the nanopipette (η = 0.001 Pa s). Therefore, μ_{eo} is 1.41 × 10^{-8} m2 /V s. $Q_{\Delta\Psi}$ at different ΔΨ are listed in:

Table S1. Electroosmotic flow at different applied potentials

$\Delta\Psi, mV$	200	400	600
$Q_{\Delta\Psi}, fL/s$	23.11	46.2	69.4

Thus, the injected volume was about 1.4 pL under +400 mV for 30 s.

The flow of molecules from a nanopipette can be written (S3):

$$J = D\nabla C + (v_p + v_{eo} + v_{ep})c \quad \text{(S3)}$$

where D is the diffusion coefficient, c is the concentration of glutamate, v_p is the pressure-induced velocity, v_{eo} is the electroosmotic velocity, and v_{ep}

is the electrophoresis-induced velocity. Since pressure was not used in this system, then $v_p = 0$. For glutamate, the charge is one in buffer solution. The following equation determines electrophoretic mobility (S4):

$$\mu_{ep} = \frac{qD}{k_B T} \quad \text{(S4)}$$

where q is the charge of molecule, k_B is the Boltzmann factor, and T is the temperature. Assuming that the diffusivity of glutamate is $4.6*10^{-10}$ m^2/s, μ_{ep} is estimated to be 1.79×10^{-8} m^2/V s.

The number of molecules that were injected onto the cell surface was (S5):

$$N = C * N_a * V = 10^{-2} * 6.02 * 10^{23} * 1{,}4 * 10^{-12} = 8.4 * 10^9\, molecules \quad \text{(S5)}$$

The concentration distribution is presented in (Figure S1) (S6):

$$\frac{c}{c_0} = (1 - e^{-\frac{Q}{4\pi DR}}) \quad \text{(S6)}$$

where c is the concentration profile out the tip of the pipette, c_0 is the concentration of molecules in the nanopipette, and Q is the flow leaving the pipette.

The calculated concentration of glutamate on the cell surface was 3.03 mM.

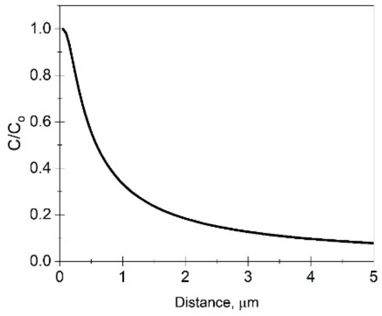

Figure S1. Concentration-distance dependence for voltage-driven glutamate delivery

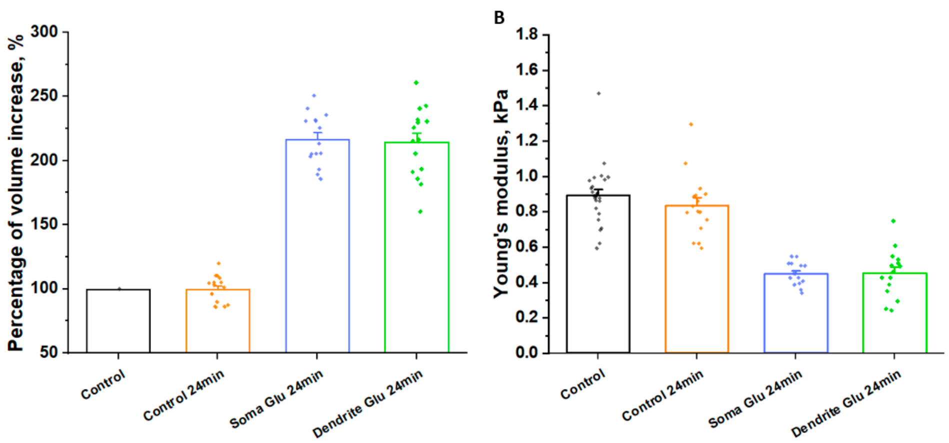

Figure S2. Mean value of cell volume increase of control cells and cells after local delivery of glutamate; (e) Mean value of Young's modulus before and after local delivery of glutamate

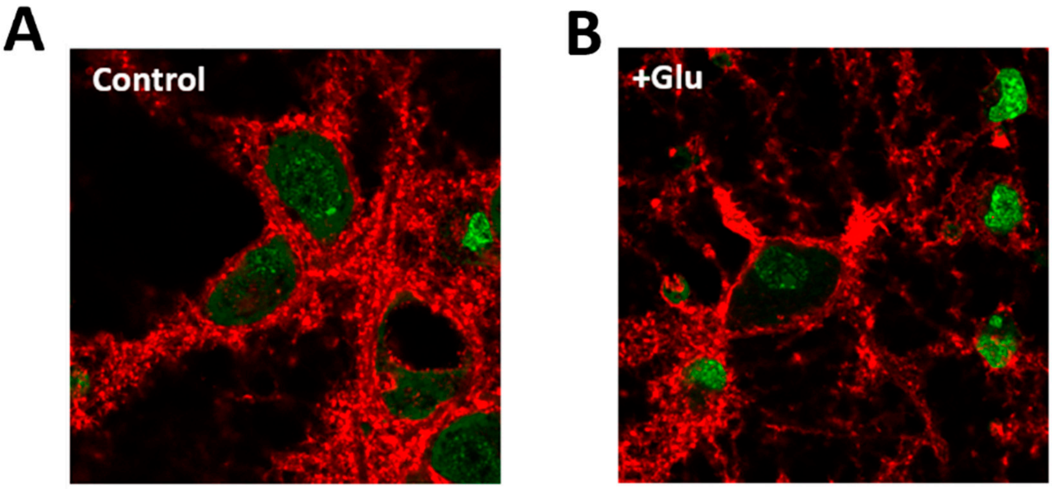

Figure S3. Confocal imaging of actin filaments of control cells (A) and cells after glutamate application (B).
